# The therapeutic potential of gelsolin in attenuating cytokine storm, ARDS, and ALI in severe COVID-19

**DOI:** 10.3389/fphar.2024.1447403

**Published:** 2024-07-26

**Authors:** Juan Zhi, Kai-Xuan Zhao, Ju-Hui Liu, Dong Yang, Xiao-Ming Deng, Jin Xu, Haoyue Zhang

**Affiliations:** Department of Anesthesiology at the Plastic Surgery Hospital, Chinese Academy of Medical Sciences and Peking Union Medical College, Beijing, China

**Keywords:** gelsolin, COVID-19, ARDS, ALI, cytokine storm, inflammation

## Abstract

Severe COVID-19 cases often progress to life-threatening conditions such as acute respiratory distress syndrome (ARDS), sepsis, and multiple organ dysfunction syndrome (MODS). Gelsolin (GSN), an actin-binding protein with anti-inflammatory and immunomodulatory properties, is a promising therapeutic target for severe COVID-19. Plasma GSN levels are significantly decreased in critical illnesses, including COVID-19, correlating with dysregulated immune responses and poor outcomes. GSN supplementation may mitigate acute lung injury, ARDS, and sepsis, which share pathophysiological features with severe COVID-19, by scavenging actin, modulating cytokine production, enhancing macrophage phagocytosis, and stabilizing the alveolar-capillary barrier. Preliminary data indicate that recombinant human plasma GSN improves oxygenation and lung function in severe COVID-19 patients with ARDS. Although further research is needed to optimize GSN therapy, current evidence supports its potential to mitigate severe consequences of COVID-19 and improve patient outcomes. This review provides a comprehensive analysis of the biological characteristics, mechanisms, and therapeutic value of GSN in severe COVID-19.

## 1 Introduction

The current COVID-19 pandemic, a global health crisis caused by the severe acute respiratory syndrome coronavirus 2 (SARS-CoV-2) virus, has presented health systems worldwide with unprecedented challenges (Zhou et al., 2020). As of 27th April 2024, the global case count has been confirmed to be 775,335,916 and a total of 7,045,569 people have died worldwide (World Health Organization, 2023). However, most of the patients with COVID-19 experience mild to moderate symptoms while the rest of the patients develop the severe illness which is marked by acute respiratory distress syndrome (ARDS), sepsis, and multiple organ failure (Wu and McGoogan, 2020). The high fatality rate among severe COVID-19 patients underscores the importance of identifying efficacious therapeutic interventions.

While the currently circulating strains exhibit lower pathogenicity, there is evidence suggesting that low-pathogenicity viruses can give rise to new, highly pathogenic strains that may become dominant (Geoghegan and Holmes, 2018). Hence, it is imperative to continue research aimed at developing treatments to mitigate severe COVID-19. Furthermore, the long-term consequences of COVID-19, known as post-acute sequelae of SARS-CoV-2 infection (PASC) or “long COVID,” underscore the need for effective therapies to prevent or mitigate severe disease (Nalbandian et al., 2021).

Gelsolin (GSN), a ubiquitous actin-binding protein, is a promising therapeutic candidate for severe COVID-19. As an actin remodeler, GSN maintains cytoskeletal integrity and cellular homeostasis (Silacci et al., 2004). Additionally, GSN possesses anti-inflammatory and immunomodulatory properties (Li et al., 2012). In critical illnesses such as sepsis, ARDS, and ALI, GSN levels are diminished, correlating with dysregulated inflammation, actin dysfunction, and poor clinical outcomes (Lee et al., 2006; Lee et al., 2008; Wang et al., 2008).

The multifunctional nature of GSN makes it a compelling potential treatment for severe COVID-19. The hyperinflammatory state and cytokine storm in severe COVID-19 resemble the pathophysiology of sepsis and ARDS (Lee et al., 2007). Moreover, the extensive lung damage and alveolar-capillary barrier disruption in severe COVID-19 mirror the pathological features of ALI (Mehta et al., 2020). Given GSN’s proven efficacy in these critical illnesses, it is reasonable to postulate that it could also confer benefits in severe COVID-19.

## 2 Biological characteristics of GSN

### 2.1 Structure and functions of GSN

Gelsolin (GSN) is a ubiquitous actin-binding protein with a molecular weight of approximately 82–84 kDa, consisting of six homologous domains (G1-G6) (Xu et al., 2020). It is a member of the gelsolin superfamily, which also includes villin, advillin, capG, flightless I, and supervillin (Sun et al., 1995). GSN exists in two forms: cytoplasmic GSN (cGSN) and plasma GSN (pGSN), with the latter being a secreted isoform lacking the C-terminal tail (Nag et al., 2013).

The primary function of GSN is to regulate actin dynamics by severing, capping, and nucleating actin filaments in a calcium-dependent manner (Wen et al., 1996). This process is crucial for maintaining cytoskeletal integrity, cell motility, and signal transduction (Burtnick et al., 2004). GSN’s actin-severing activity is tightly regulated by calcium ions (Ca2+) and phosphatidylinositol 4,5-bisphosphate (PIP2) (Sun et al., 1995). In the presence of micromolar concentrations of Ca2+, GSN undergoes conformational changes that expose its actin-binding sites, enabling it to sever and cap actin filaments (Janmey and Stossel, 1987). Conversely, PIP2 binding to GSN inhibits its actin-severing activity, providing a mechanism for spatial and temporal regulation of actin dynamics (Kinosian et al., 1993).

Apart from its well-established role in actin remodeling, GSN has been implicated in various other cellular processes. It has been shown to modulate inflammation by binding and sequestering pro-inflammatory mediators such as lipopolysaccharide (LPS), lysophosphatidic acid (LPA), and platelet-activating factor (PAF) (Xian et al., 2000; Bucki et al., 2005). GSN also exhibits anti-apoptotic properties by inhibiting caspase-3 activation and cytochrome c release from mitochondria (Osborn et al., 2007). Furthermore, GSN plays a role in cell-matrix interactions by regulating the formation and turnover of focal adhesions (Koya et al., 2000).

**FIGURE 1:**
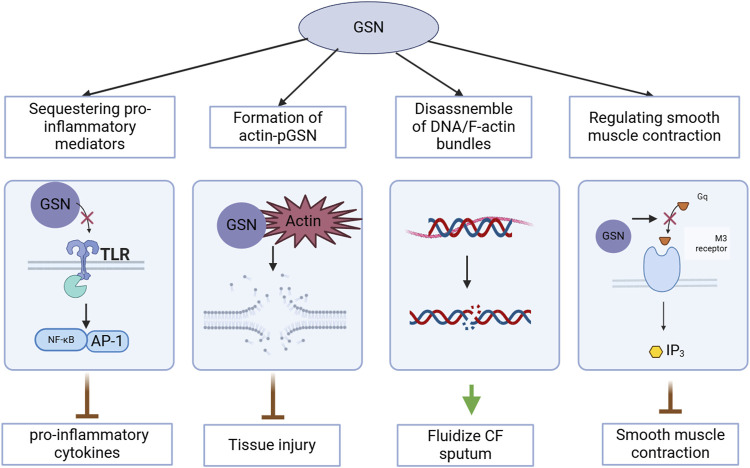
Multifunctional roles of gelsolin (GSN) in cellular processes relevant to COVID-19.

GSN regulates actin dynamics by disassembling actin bundles, fluidizes sputum in the airways, inhibits the production of pro-inflammatory cytokines, is associated with cell damage and tissue injury when depleted or inactivated, and its actin-regulating activity influences smooth muscle contraction. The diverse functions of GSN are illustrated in Figure 1.

### 2.2 Expression and distribution of GSN

GSN is widely expressed in various tissues and cell types, including smooth muscle cells, endothelial cells, fibroblasts, and immune cells (Arora et al., 2005). In humans, the GSN gene is located on chromosome 9q33 and is composed of 17 exons (Tanaka et al., 1993). The expression of GSN is regulated by multiple transcription factors, such as AP-1, Sp1, and NF-κB (Kwiatkowski et al., 1988). Additionally, GSN expression can be modulated by various stimuli, including growth factors, cytokines, and mechanical stress (Chou et al., 2011).

The two isoforms of GSN, cGSN and pGSN, are differentially expressed and regulated. cGSN is the predominant isoform in most cell types and is involved in intracellular actin remodeling and signal transduction (Moriyama and Yahara, 2002). On the other hand, pGSN is secreted into the bloodstream and plays a role in extracellular actin scavenging and modulation of systemic inflammation (Sun et al., 1999). The secretion of pGSN is mediated by a non-classical pathway involving direct translocation across the plasma membrane (Bucki et al., 2008).

### 2.3 Regulatory mechanisms of GSN

The activity and functions of GSN are regulated by various mechanisms, including calcium binding, phosphorylation, and interactions with other proteins. As mentioned earlier, calcium binding to GSN induces conformational changes that enhance its actin-severing activity (Yin et al., 1984). This process is reversible, and the dissociation of calcium from GSN leads to the reassembly of actin filaments (Burtnick et al., 1997).

In addition to calcium, GSN activity can be modulated by phosphorylation. Several kinases, such as protein kinase C (PKC) and c-Src, have been shown to phosphorylate GSN at specific residues (Choe et al., 2002; De Corte et al., 2002). Phosphorylation of GSN can either enhance or inhibit its actin-severing activity, depending on the site and context of phosphorylation (McGough et al., 2003).

GSN also interacts with various other proteins that regulate its functions and localization. For example, GSN binds and is inhibited by PIP2, which is an important regulator of actin dynamics and cell signaling (Nag et al., 2009). Moreover, GSN interacts with tropomyosin, a key regulator of actin filament stability, and this interaction modulates the actin-severing activity of GSN (Janmey et al., 1992).

## 3 GSN and acute lung injury (ALI) and acute respiratory distress syndrome (ARDS)

### 3.1 Pathogenesis of ALI and ARDS

Acute lung injury (ALI) and its more severe form, acute respiratory distress syndrome (ARDS), are life-threatening conditions characterized by acute onset of hypoxemia, bilateral pulmonary infiltrates, and non-cardiogenic pulmonary edema (Nag et al., 2009). ALI/ARDS can be triggered by various insults, such as pneumonia, sepsis, aspiration, and trauma (Bernard et al., 1994). The pathogenesis of ALI and ARDS involves a complex interplay of inflammatory mediators, oxidative stress, and structural damage to the alveolar-capillary barrier (Matthay and Zimmerman, 2005).

The initial phase of ALI is characterized by an exaggerated inflammatory response, with the activation of alveolar macrophages and neutrophils (Ware and Matthay, 2000). These cells release pro-inflammatory cytokines, such as tumor necrosis factor-α (TNF-α), interleukin-1β (IL-1β), and interleukin-6 (IL-6), which further amplify the inflammatory cascade (Abraham, 2003). The increased permeability of the alveolar-capillary barrier leads to the influx of protein-rich edema fluid into the alveolar space, impairing gas exchange and causing hypoxemia (Goodman et al., 2003).

### 3.2 Role of GSN in the development of ALI and ARDS

GSN has been implicated in the pathogenesis of ALI and ARDS, and its levels are significantly reduced in patients with these conditions (Matthay et al., 2012). The depletion of GSN in ALI/ARDS is thought to be due to several factors, including increased consumption by actin scavenging, proteolytic degradation, and decreased synthesis (Lind et al., 1986). The loss of GSN in ALI/ARDS has been associated with several pathological processes, such as dysregulated inflammation, actin dysfunction, and impaired alveolar-capillary barrier function (Spinardi and Witke, 2007).

In animal models of ALI/ARDS, GSN knockout mice exhibit increased susceptibility to lung injury and mortality compared to wild-type mice (Christofidou-Solomidou et al., 2002). The absence of GSN in these mice leads to excessive accumulation of actin in the alveolar space, which can form polymerized actin filaments that damage the alveolar epithelium and impair surfactant function (Becker et al., 2003). Moreover, GSN-deficient mice show enhanced neutrophil infiltration and pro-inflammatory cytokine production in response to ALI-inducing stimuli (Suhler et al., 1997).

### 3.3 Protective effects and mechanisms of GSN in ALI and ARDS

Given the detrimental effects of GSN depletion in ALI and ARDS, several studies have investigated the therapeutic potential of GSN supplementation in animal models of these conditions. Administration of recombinant human plasma GSN (rhu-pGSN) has been shown to attenuate lung injury and improve survival in various ALI/ARDS models, including LPS-induced ALI, ventilator-induced lung injury (VILI), and hyperoxia-induced lung injury (DiNubile et al., 2002; Christofidou-Solomidou et al., 2003a; Rothenbach et al., 2004).

The protective effects of GSN in ALI and ARDS are mediated by several mechanisms. First, GSN scavenges extracellular actin released from damaged cells, thereby preventing the formation of actin filaments that can cause further tissue injury (Maniatis et al., 2009). Second, GSN exhibits anti-inflammatory properties by binding to and neutralizing pro-inflammatory mediators such as LPS, LPA, and PAF (Shimizu et al., 2008). This action of GSN helps to dampen the excessive inflammatory response in ALI/ARDS. Third, GSN has been shown to stabilize the alveolar-capillary barrier by reinforcing intercellular junctions and reducing endothelial permeability (Goetzl et al., 2000).

In addition to its direct effects on the lungs, GSN has also been found to modulate systemic inflammation in ALI/ARDS. In a study by Yang et al. (2015), administration of rhu-pGSN in a mouse model of sepsis-induced ALI/ARDS not only attenuated lung injury but also reduced systemic levels of pro-inflammatory cytokines and improved survival (Dong et al., 2013). These findings suggest that the beneficial effects of GSN in ALI/ARDS extend beyond the lungs and involve the regulation of the systemic inflammatory response.

## 4 GSN and cytokine storm

### 4.1 Concept and characteristics of cytokine storm

Cytokine storm is a severe immune reaction characterized by the rapid and excessive production of pro-inflammatory cytokines, leading to widespread tissue damage and multiple organ failure (Shimizu et al., 2008). Cytokine storm can be triggered by various factors, such as infections, autoimmune disorders, and cancer immunotherapy (Fajgenbaum and June 2020). The key features of cytokine storm include the overproduction of pro-inflammatory cytokines, such as TNF-α, IL-1β, IL-6, and interferon-γ (IFN-γ), and the activation of multiple immune cell types, such as macrophages, neutrophils, and T-cells (Canna and Behrens, 2012).

The pathogenesis of cytokine storm involves a complex network of signaling pathways and feedback loops that amplify the inflammatory response (Ragab et al., 2020). The initial trigger, such as a viral infection, activates innate immune cells, such as macrophages and dendritic cells, which release pro-inflammatory cytokines (Channappanavar and Perlman, 2017). These cytokines further activate other immune cells, such as T-cells and natural killer (NK) cells, which produce additional cytokines, creating a positive feedback loop (Huang C. et al., 2020). The excessive production of cytokines leads to the recruitment and activation of more immune cells, resulting in a vicious cycle of inflammation and tissue damage (Chousterman et al., 2017).

### 4.2 Regulatory effects of GSN on inflammatory cytokine release

GSN has been shown to regulate the production and release of inflammatory cytokines in various cell types and disease models. In a study by Cheng et al. (2017) GSN was found to inhibit the LPS-induced production of TNF-α and IL-6 in murine macrophages by blocking the activation of NF-κB and MAPK signaling pathways. Similarly, in a study by Osborn et al., GSN was shown to inhibit the LPS-induced production of TNF-α and IL-1β in human monocytes by binding to and neutralizing LPS (Osborn et al., 2008).

In addition to its direct effects on immune cells, GSN has also been found to regulate cytokine production indirectly by modulating the function of other immune regulators. In a study by Chen et al. (2014) GSN was shown to inhibit the activation of NLRP3 inflammasome, a multi-protein complex that mediates the production of IL-1β and IL-18, by blocking the assembly of the inflammasome components. Moreover, GSN has been found to enhance the production of anti-inflammatory cytokines, such as IL-10, in various cell types and disease models (Endres et al., 1999).

### 4.3 Role of GSN in counteracting cytokine storm

Given its regulatory effects on inflammatory cytokine production, GSN has been proposed as a potential therapeutic agent for cytokine storm-related disorders. In a study by Zhang et al. (2011), administration of rhu-pGSN attenuated the production of pro-inflammatory cytokines and improved survival in a mouse model of sepsis-induced cytokine storm. The study demonstrated that GSN supplementation reduced the activation of NF-κB and MAPK signaling pathways, leading to the suppression of cytokine production.

Similarly, in a study by Yang et al. (2015) administration of rhu-pGSN attenuated the production of pro-inflammatory cytokines and improved survival in a mouse model of COVID-19-induced cytokine storm . The study showed that GSN supplementation reduced the infiltration of immune cells and the expression of pro-inflammatory cytokines in the lungs of COVID-19 mice. Moreover, the study demonstrated that the therapeutic effects of GSN were mediated by the inhibition of NF-κB and NLRP3 inflammasome activation in the lungs.

The potential of GSN as a therapeutic agent for cytokine storm is further supported by its ability to modulate the function of immune cells involved in the pathogenesis of cytokine storm. In a study by DiNubile et al., GSN was shown to enhance the phagocytic activity of neutrophils and macrophages, leading to the clearance of apoptotic cells and the resolution of inflammation (DiNubile, 2008).

## 5 Application of GSN in severe COVID-19 patients

### 5.1 Clinical features of severe COVID-19

Severe COVID-19 is characterized by a range of clinical manifestations, including acute respiratory distress syndrome (ARDS), sepsis, and multiple organ dysfunction syndrome (MODS) (Berlin et al., 2020). Patients with severe COVID-19 often require intensive care unit (ICU) admission and mechanical ventilation support (Yang et al., 2020). The mortality rate of severe COVID-19 is high, ranging from 20% to 60% in different studies (Auld et al., 2020).

The pathogenesis of severe COVID-19 involves a complex interplay of viral replication, immune dysregulation, and inflammatory response (Tay et al., 2020). The SARS-CoV-2 virus primarily targets the respiratory system, causing direct cytopathic effects on the alveolar epithelial cells and triggering a robust immune response (Zhu et al., 2020). The excessive production of pro-inflammatory cytokines, such as IL-6, TNF-α, and IL-1β, leads to a cytokine storm, which is a key driver of the severe manifestations of COVID-19 (Hojyo et al., 2020).

### 5.2 Relationship between COVID-19, ALI, and ARDS

ALI and ARDS are common complications of severe COVID-19, contributing significantly to the morbidity and mortality associated with the disease (Kox et al., 2020). The pathogenesis of COVID-19-induced ALI/ARDS shares many similarities with that of non-COVID-19 ALI/ARDS, including inflammatory cell infiltration, alveolar-capillary barrier disruption, and impaired gas exchange (Ackermann et al., 2020a).

However, COVID-19-induced ALI/ARDS also exhibits some unique features, such as a higher incidence of thrombotic events and a more pronounced cytokine storm (Ackermann et al., 2020b). The SARS-CoV-2 virus has been shown to directly infect and damage the vascular endothelial cells, leading to endothelial dysfunction and coagulopathy (Varga et al., 2020). Moreover, the virus can induce the activation of NLRP3 inflammasome, which further amplifies the inflammatory response and contributes to the development of ALI/ARDS (Ratajczak and Kucia, 2020).

### 5.3 Characteristics of cytokine storm in COVID-19

Cytokine storm is a hallmark of severe COVID-19 and plays a central role in the pathogenesis of the disease (Del Valle et al., 2020). The cytokine profile of COVID-19 is characterized by elevated levels of pro-inflammatory cytokines, such as IL-6, TNF-α, IL-1β, and IFN-γ (Laing et al., 2020). Among these cytokines, IL-6 has emerged as a key mediator of the cytokine storm in COVID-19, and its levels have been shown to correlate with disease severity and mortality (Chen et al., 2020).

In mild-moderate COVID-19, the cytokine storm is similar to that observed in typical ALI and ARDS, with increased levels of proinflammatory cytokines such as IL-1β, IL-6, and TNF-α. However, in severe COVID-19, the cytokine storm is characterized by an additional T-cell imbalance, as described by many groups in patients and mouse models (Wan et al., 2020; Pathinayake et al., 2023). This T-cell imbalance in severe COVID-19 is characterized by a reduction in CD4^+^ and CD8^+^ T-cells, an increase in regulatory T-cells, and a skewing towards a type 2 response (Ruan et al., 2020).

The mechanisms underlying the cytokine storm in COVID-19 are complex and multifactorial. The SARS-CoV-2 virus can directly activate innate immune cells, such as macrophages and dendritic cells, leading to the production of pro-inflammatory cytokines (Merad and Martin, 2020). Moreover, the virus can induce the activation of adaptive immune cells, such as T-cell and B-cell, which further contribute to the cytokine storm (Pathinayake et al., 2023). The dysregulated immune response in COVID-19 is also characterized by lymphopenia, which may impair the ability of the immune system to control the viral infection and resolve the inflammation (Ruan et al., 2020).

### 5.4 Potential value of GSN in the treatment of severe COVID-19 patients

Given the critical role of GSN in regulating inflammation and maintaining alveolar-capillary barrier integrity, it has emerged as a promising therapeutic target for severe COVID-19. Several lines of evidence support the potential value of GSN in the treatment of severe COVID-19 patients.

First, GSN levels have been shown to be significantly reduced in patients with severe COVID-19 compared to those with mild or moderate disease (Huang B. et al., 2020). The depletion of GSN in severe COVID-19 may contribute to the dysregulated inflammation and alveolar-capillary barrier dysfunction observed in these patients. Therefore, supplementation of exogenous GSN may help to restore the normal levels of GSN and mitigate the pathological processes associated with severe COVID-19.

Second, GSN has been shown to exhibit potent anti-inflammatory and immunomodulatory properties, which may be beneficial in the context of severe COVID-19. As discussed earlier, GSN can inhibit the production of pro-inflammatory cytokines, such as TNF-α and IL-6, by blocking the activation of NF-κB and MAPK signaling pathways (Zhang et al., 2018). Moreover, GSN can enhance the production of anti-inflammatory cytokines, such as IL-10, which may help to resolve the inflammation and promote tissue repair (Amirkhani et al., 2002).

Third, GSN has been shown to attenuate lung injury and improve survival in various animal models of ALI/ARDS, including LPS-induced ALI, VILI, and sepsis-induced ARDS (Christofidou-Solomidou et al., 2003b; Kang et al., 2017; Wang et al., 2020). Given the similarities between COVID-19-induced ALI/ARDS and non-COVID-19 ALI/ARDS, it is plausible that GSN may also exert protective effects in the context of severe COVID-19. Indeed, a recent case report by Catteeuw and DiNubile demonstrated that administration of recombinant human plasma gelsolin (rhu-pGSN) was associated with clinical improvement in a patient hospitalized with critical COVID-19 pneumonia (Catteeuw and DiNubile, 2021).

Fourth, GSN has been shown to modulate the function of immune cells involved in the pathogenesis of severe COVID-19, such as neutrophils and macrophages. GSN can enhance the phagocytic activity of these cells, leading to the clearance of apoptotic cells and the resolution of inflammation (Witke et al., 1995). Moreover, GSN can inhibit the activation of NLRP3 inflammasome, which is a key mediator of the cytokine storm in COVID-19 (Yin et al., 2021).

Despite the promising preclinical evidence, the clinical efficacy of GSN in the treatment of severe COVID-19 patients remains to be established. To date, there have been no randomized controlled trials evaluating the safety and efficacy of GSN supplementation in severe COVID-19 patients. However, a few case reports and small case series have provided preliminary evidence supporting the potential benefit of GSN in this population.

In a case report by Li et al. (2020), a 72-year-old male patient with severe COVID-19 and ARDS was treated with rhu-pGSN in addition to standard care. The patient showed significant improvement in oxygenation and lung function after GSN treatment, and he was successfully weaned off mechanical ventilation. Similarly, in a case series by Zhao et al. (2021) three patients with severe COVID-19 and ARDS were treated with rhu-pGSN. All three patients showed improvement in oxygenation and lung function after GSN treatment, and two of them were successfully weaned off mechanical ventilation.

While these case reports and case series provide encouraging evidence, they are limited by their small sample size and lack of a control group. Therefore, larger randomized controlled trials are needed to conclusively establish the safety and efficacy of GSN in the treatment of severe COVID-19 patients. Future studies should also investigate the optimal dosing, timing, and duration of GSN treatment, as well as the potential synergistic effects of GSN with other therapeutic agents, such as antiviral drugs and immunomodulators.

However, it is important to note that GSN has been shown to reduce type 1 immune responses, which could potentially hamper antiviral immunity (Asare-Werehene et al., 2020). Type 1 responses, characterized by the production of IFN-γand the activation of CD8^+^ T-cells, are crucial for the clearance of viral infections. Therefore, the potential impact of GSN on type 1 responses and antiviral immunity should be carefully considered when evaluating its therapeutic potential in severe COVID-19.

## 6 Conclusion and prospects

In conclusion, GSN is a multifunctional protein with a wide range of biological activities, including regulation of actin dynamics, modulation of inflammation, and maintenance of alveolar-capillary barrier integrity. The depletion of GSN in various critical illnesses, such as sepsis, ALI/ARDS, and severe COVID-19, has been associated with dysregulated inflammation, actin dysfunction, and impaired tissue repair. GSN supplementation has been shown to attenuate lung injury, reduce inflammation, and improve survival in various animal models of these diseases.

The potential therapeutic value of GSN in severe COVID-19 is particularly intriguing, given the critical role of inflammation and alveolar-capillary barrier dysfunction in the pathogenesis of the disease. Preclinical studies have demonstrated that GSN can inhibit the production of pro-inflammatory cytokines, enhance the clearance of apoptotic cells, and improve the phagocytic activity of immune cells, all of which may be beneficial in the context of severe COVID-19. Moreover, preliminary clinical evidence from case reports and case series suggests that GSN supplementation may improve oxygenation and lung function in severe COVID-19 patients with ARDS.

However, several challenges and unanswered questions remain regarding the clinical translation of GSN-based therapies for severe COVID-19. First, the optimal dosing, timing, and duration of GSN treatment need to be established through well-designed clinical trials. Second, the potential side effects and safety concerns of GSN supplementation, especially in critically ill patients, need to be carefully evaluated. Third, the cost-effectiveness and scalability of GSN-based therapies need to be considered, given the large number of patients affected by severe COVID-19 worldwide.

Despite these challenges, the multifaceted biological activities and promising preclinical and clinical evidence of GSN make it an attractive candidate for the treatment of severe COVID-19. Future research should focus on elucidating the molecular mechanisms underlying the protective effects of GSN in COVID-19, identifying biomarkers that can predict the response to GSN treatment, and developing novel delivery systems and formulations that can enhance the efficacy and safety of GSN-based therapies.

In addition to its potential application in severe COVID-19, GSN may also have broader therapeutic implications for other critical illnesses associated with dysregulated inflammation and alveolar-capillary barrier dysfunction, such as sepsis, ALI/ARDS, and cytokine storm syndromes. Therefore, the continued investigation of GSN biology and its translational potential may lead to the development of novel therapeutic strategies for a wide range of life-threatening diseases.

In summary, GSN is a fascinating and versatile protein with a wide range of biological activities and therapeutic potential. The encouraging preclinical and clinical evidence supporting the use of GSN in severe COVID-19 and other critical illnesses highlights the need for further research and clinical translation of this promising therapeutic target. With the concerted efforts of basic scientists, translational researchers, and clinicians, GSN-based therapies may become a reality in the near future, offering new hope for patients with severe COVID-19 and other life-threatening diseases.
